# Chinese Medicine Phenomics (Chinmedphenomics): Personalized, Precise and Promising

**DOI:** 10.1007/s43657-022-00074-x

**Published:** 2022-10-14

**Authors:** Chunchun Yuan, Weiqiang Zhang, Jing Wang, Chen Huang, Bing Shu, Qianqian Liang, Tingrui Huang, Jiucun Wang, Qi Shi, Dezhi Tang, Yongjun Wang

**Affiliations:** 1 Longhua Hospital, Shanghai University of Traditional Chinese Medicine, Shanghai, 200032 China; 2 Institute of Spine, Shanghai University of Traditional Chinese Medicine, Shanghai, 200032 China; 3 Key Laboratory of the Ministry of Education about Theory and Treatment of Muscles and Bones, Shanghai, 200032 China; 4 State Key Laboratory of Genetic Engineering, Collaborative Innovation Center for Genetics and Development, School of Life Sciences and Human Phenome Institute, Fudan University, Shanghai, 200433 China; 5 Academic Research Center of Shixiaoshan’ Traumatology, Shanghai, 200032 China; 6 Famous Traditional Chinese Medicine Office, Shanghai, 200032 China

**Keywords:** Chinese medicine phenomics (Chinmedphenomics), Mega cohort, Multi-disciplinary association, Precision medicine

## Abstract

The systematicness of phenomics and Traditional Chinese Medicine (TCM) enable these two disciplines to interlink with each other. This article discussed the similarity in theory and application between TCM and phenomics and illustrates their respective advantages in diagnosis and treatment of diseases, forming a new discipline eventually. Chinese medicine phenomics (Chinmedphenomics) is built on classic TCM, combined with phenomics technology, and the development of which needs the mega cohort with TCM syndrome and the characteristics of precision medicine as well as multi-disciplinary cooperation, which is personalized, precise and promising, providing unique scientific insights into understanding human health.

## Introduction

Phenomics is an emerging discipline that analyzes how the environment interacts with the expression of genes (Houle et al. 2010). Basically, it is defined as a set of measurable traits, including the physical, chemical, and biological traits of individuals and populations, resulting from complex interactions between genes and environment, which includes molecular microenvironment (Jin 2021).

**Fig. 1:**
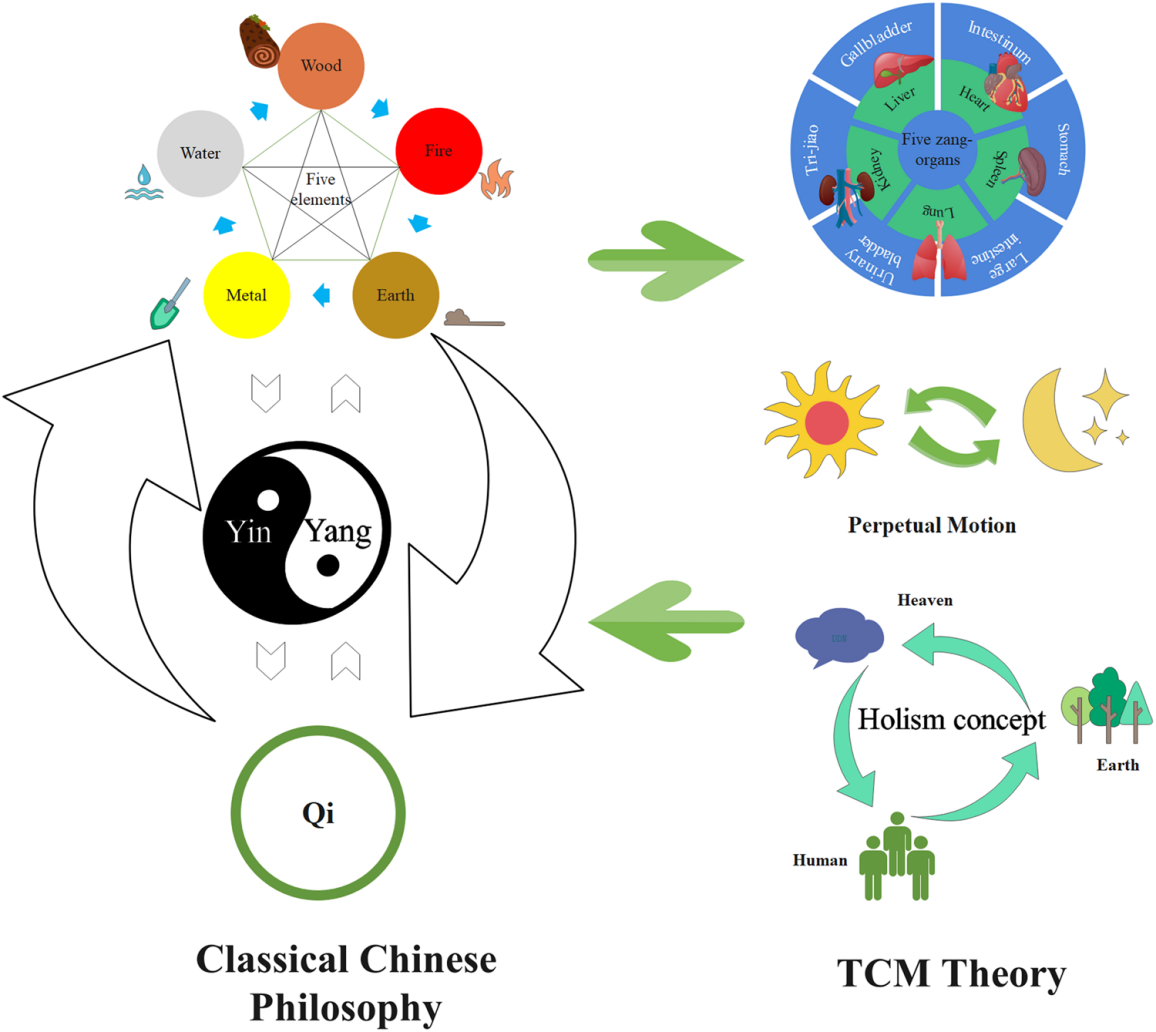
Relationship between classic Chinese philosophy and TCM. TCM, based on classical Chinese philosophy theories including qi, yin-yang, and five elements theory, focuses on heaven, earth, humans, and their interrelations, innovating and developing the standpoints of five zang-organs theory, perpetual motion, and holism concept

Traditional Chinese Medicine (TCM) is the crystallization of Chinese wisdom, based on classical Chinese philosophy theories including essence, qi (pneuma), yin-yang and five elements theory, aiming at storing and restoring human healthy, guided by the methodology of a holistic and systematic view (holism concept). Essence, changing along the whole process of life, is becoming sufficient since birth and reaching the peak when people are 20–30 years old, as the driving force of human growth (Shu et al. 2015). Qi (pneuma), a very fine substance, is one of the primary substances forming body and driving forces of biological activities (Leong et al. 2015). Yin-yang balance is used to summarize two opposite aspects of interrelated things. However, yin and yang have complicated relationship. They are opposed to and, at the same time, depend on each other (Yan et al. 2020). The five elements refer to wood, fire, earth, metal, and water as well as their motion and change, and everything can be attributed to one of them, having mutual interaction among them (Pun and Chor 2022). Actually, these theories focusing on heaven, earth, human, and their interrelations, innovating and developing the standpoints of five zang-organs, perpetual motion, and concept of holism (Fig. 1). Over 2000 years, TCM has established systematic medical theories about disease diagnosis and treatment, even humanistic care, with its own unique theoretical system (Hao et al. 2011). From its origin, TCM has been equipped with phenomics content. It is believed that TCM combined with phenomics technology, may promote formation of new phenomics with Chinese characteristics, thus further developing TCM, and taking full advantage of it.

## Methods of Understanding Things in TCM Share Similarities with Phenomics

TCM approaches to understanding the world share similarities with phenomics. They emphasize the correspondence between human and nature, sharing an idea that humans are rooted in nature, being subject to natural and environmental changes including climate, district, and diet (Fig. 1). TCM is based on yin and yang principle, i.e., good health is believed to come from a balance of yang (positive) and yin (negative) (Fu et al. 2021). Climate changes including wind, cold, summer heat, dampness, dryness, and fire, can affect normal physical function (Hai-Long et al. 2015). For example, wind affects the upper part of the human body, such as the head and lung, which correspondingly have the characteristics of yang. Dampness usually affects the spleen and has the characteristics of yin. Yin and yang may reject and attract each other, respectively. Especially for the internal organs of the body, the yin or yang property of them makes themselves always easily suffer from the corresponding pathogenic factors in pathological conditions. Besides, TCM emphasized the importance of unity of body and spirit, underlining the balance between biological property and spiritual consciousness property in humans (Wan et al. 2012). Qi, blood, and body fluid are all the essential substance to form the body and maintain life activities with a strong vitality and continuous movement in the human body, having a mediating role in connecting body and spirit. Disturbances in body or spirit usually contribute to a corresponding imbalance of spirit or body by qi, blood, and body fluid (Yao et al. 2013).

TCM equally focuses on inborn and acquired characteristics of the human body. Essence represents inborn qualities, which might be equivalent to a gene or stem cells. According to the inborn and acquired theory, inborn essence, mainly conferred by parents, dominates the major aspects of life, including lifetime, reproductive function, mentality level, and prognosis (Li et al. 2019). However, these aspects may also be affected by acquired essence, produced by viscera, mainly spleen and stomach, extracting and purifying food and water. Thus, it may be concluded that TCM enriches its own phenomics cognition by observing large numbers of external and internal phenotypes.

## TCM Treating Life Phenomena Is Personalized

The holism concept of TCM is majorly reflected in the following aspects. First, it connects humans with nature. Humans are affected by nature, such as earth, climate, season, temperature, humidity, etc. Humans also exert subjective initiative to accommodate and remold nature to some degree. Second, viscera in the internal part and external syndromes are connected. “Viscera inside the body must manifest themselves externally.” Internal visceral disorders cause external syndromes. For example, a doctor can identify pathological organs and pathological stages by analyzing the external syndromes, including pulse, tongue, smell, and other syndromes. All these are phenotypes, also known as Chinmedphenomics. On the other hand**,** persistent external influence of bad lifestyle or surroundings may lead to visceral function imbalance or degradation and pathological condition. Syndrome differentiation and treatment are the most distinctive feature of the holistic approach of TCM.

According to the TCM principle, the syndrome is a professional term that summarizes disease′s unique characteristics and development regulation (Mei 2011). Syndrome, including symptoms and signs, is the pathological generalization of a certain period or a certain type of pathology in the disease process. A syndrome or pattern, also known as Zheng, is the key concept of TCM theory that is considered for further stratification of patients when it is integrated with biomedical diagnoses in clinical practice (Zhang et al. 2013). A doctor makes a summary of the essence based on the etiologies, the locus and properties of the disease, the pathogenesis, the patient constitution, the inborn endowment, and even the psychologic situation, giving a comprehensive and intrinsic judgment for the symptom and characteristics of diseases. The reasons for “treating the same disease with different therapies and treating the different diseases with the same therapies” are just explained by the syndrome differentiation and treatment (Zhang et al. 2022), thus making syndrome differentiation the focus of TCM treatment.

Phenomics emphasizes the interactions between genes and environment, and the molecular mechanisms are greatly involoved, which is mostly in accordance with the TCM explorement of life phenomena and diseases.

## TCM Prescription in Disease Treatment Is Precise

**Fig. 2:**
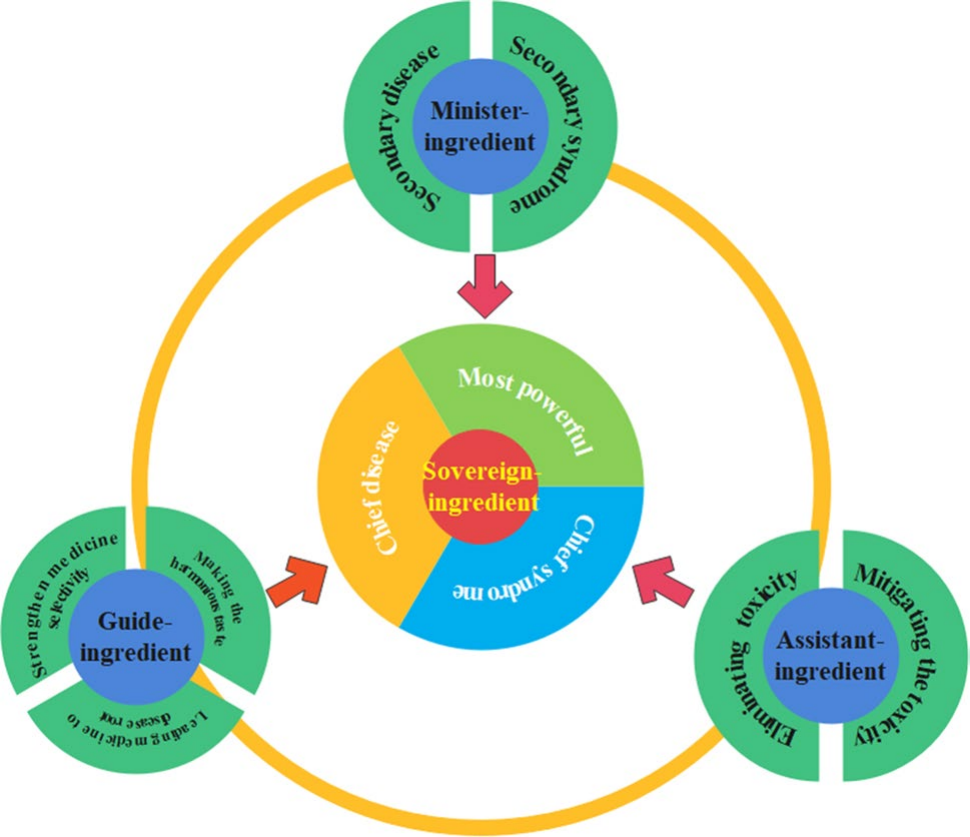
Concept of TCM prescription, which consists of the ingredients of Sovereign, Minister, Assistant, and Guide

As one of the major therapeutic methods in clinic, Chinese medicinal prescriptions are written based on the ingredients of sovereign, minister, assistant, and guide (Fig. 2). Sovereign herbs are used to treat the serve diseases or syndromes, with strong medicinal effect. Minister herbs have synergistic effects with Sovereign herbs, which help alleviate other accompanying symptoms. Assistant herbs enhance the therapeutic effects and modulate the adverse effects of sovereign and minister herbs. The guide herbs include two kinds of meaning, leading the rest of herbal medicine directly to disease position or keeping the herbal medicine in the prescription in a harmonious status, usually without excessively cold or hot properties (Wu et al. 2012). Phenomics suggests that treating diseases and disorders could consider the integrative perspectives of intrinsic disease-causing mechanisms and correspondingly external manifestation. Intrinsic disease-causing factors include disorders of metabolism, signaling channel, and even gene expression regulation (Brookes and Robinson 2015).

Taking into account the chain reactions or multidirectional contacts that occur in the human body during the occurrence of diseases, phenomics emphasizes precise treatment, which is never a unidirectional treatment. Treatments should consider the lesion target organs, disordered target signaling channels, impaired core metabolism mechanism, and even the affected nervous central system (Maiella et al. 2018). Chinese medical prescription, with syndrome differentiation and treatment and holism concept, might solve the problem. Acupuncture, cupping, tuina, and other TCM treatment measures also embody the phenomics by meridian system or midnight-noon ebb-flow theory.

## Chinmedphenomics Formed by Combining TCM with Modern Technology Is Promising

**Fig. 3:**
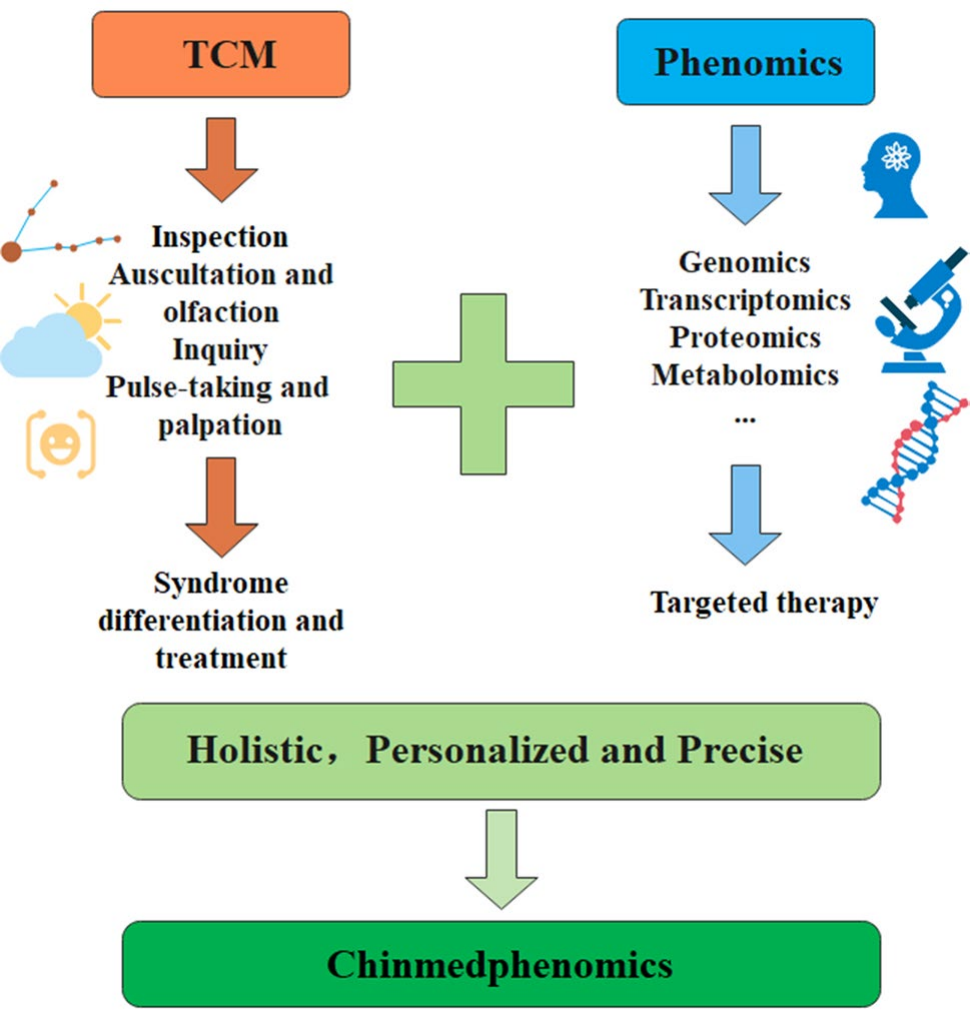
Overview of TCM, phenomics and Chinmedphenomics

Chinmedphenomics, has a positive impact on TCM and phenomics, promoting the development of both TCM and phenomics (Fig. 3). On the one hand, the concept of TCM has an enlightening effect on the development of phenomics. TCM investigates the correlations between external expressions and the internal pathological mechanism based on the syndrome's characteristics, providing a reference for tracking the disease process and determining the causes of diseases in different stages (Zhang et al. 2013). Whole-genome sequencing, proteome, and metabolome are widely used in phenomics to construct the diseases-related network (Wang et al. 2021a) or find the potential relations between external expressions and internal genes, proteins, or molecular metabolism alteration (Wang et al. 2021b), and yet these methods are costly, complex, and may lead to incomplete and insufficient data due to the complexity of life activities. Investigating the potential syndrome causes of diseases and analyzing the human body microenvironment regulations with the syndrome changing may advance our understanding of the diseases from the prespective of phenomics.

On the other hand, phenomics could greatly enrich the diagnosis and treatment technology of TCM. Muti-omics measurement, one of phenomics technologies that aims at molecule levels, enables finding specific targets, including special genes, receptors, enzymes, or signaling channels (Ye et al. 2020), which is exactly what TCM needs. Taking five elements theory of the five zang organs of TCM as an example, the organ′s function and susceptibility with external surroundings have been integrated from macroscopic perspectives, e.g., Kidney governs water, storing essence and manufacturing marrow, opening into the ears, anus, and urethra, easily suffering from coldness and deficiency of kidney-essence, and always affecting lung function by not receiving qi. Although all the regular summary and understanding of humans and nature in TCM provides rich phenotypes and interactions, forming the solid theoretical and practical foundation for primary Chinmedphenomics, potential mechanisms for the interactions and correlations mentioned above in TCM need to be investigated at the molecular level with omics technologies to reclassify Chinmedphenomics.

## Current Developments and Prospects of Chinmedphenomics

Many studies have been carried out to investigate the intrinsic molecular mechanism or potential causes of TCM syndrome, seeking the bridge for connecting syndromes and internal pathologic mechanism. Different proteins and vitamins could contribute to the syndromes of chronic heart failure patients, including qi deficiency, blood stasis, and yin deficiency. For example, patients with osteoporosis tend to suffer from spleen and kidney yang deficiency or liver and kidney yin deficiency syndrome. Also, middle-elder people with yang deficiency constitution often develop osteopenia, which is believed to be related to DBP4588 single nucleotide polymorphism, an important vitamin D binding protein gene locus that can affect vitamin D level and bone mineral density levels (Powe et al. 2011). Aging is a permanent topic for medical research concerning metabolism changes, genes, and telomerase. According to TCM, it is the inborn essence in the kidney that dominates the life span and activities. Serum creatinine, mainly produced by creatine in muscle, is excreted at a constant rate through the kidney daily, always regarded as a marker of kidney function (Rahn et al. 1999). According to the *Plain Question*, a classic ancient theory of TCM, bad complexion and white hair usually appear at 42 years old for women and 48 years old for men as the obvious aging phenotypes, which are always considered to be caused by gradually consumed essence in the kidney. So, serum creatinine might not only a biomarker for evaluating kidney function but also more probably an objective evaluation index of kidney essence, casting beneficial inspiration on the Chinmedphenomics research.

Chinmedphenomics development needs a large study cohort with TCM syndrome and disease characteristics. In the past, individual medical records and experience summaries are the main research contents for TCM, for which long-term follow-up is impossible and the scientific breakthroughs for precision medicine and healthcare are hardly discovered in time. In TCM, the holism concept and the correspondence between nature and human theories emphasize the medical model of society–psychology–physiology, which corresponds to the merits of the mega population cohort. Mega population cohort can recruit enough positive cases to investigate the fundamental correlations between genetic factors and the environment associated with diseases. The China Community-based Cohort of Osteoporosis (CCCO) is a large-scale Chinmedphenomics cohort (Wang et al. 2019). The project aims to collect Chinmedphenomics data at various scales of genetics, environment, and lifestyle. It will be carried out in seven major provinces (regions) across the country, including Beijing, Shanghai, Jiangxi province, Guangdong province, Jilin province, Gansu province, and Henan province, and approximately 200,000 people will be involved in the project, with multi-factor analysis, multi-disciplinary cooperation, and prospective cohort study of multi-diseases, including chronic diseases with TCM characteristics.

Multi-disciplinary cooperation and machine learning are the major methods for exploring the underlying mechanisms of Chinmedphenomics. No matter physiological or pathological procedures are all concerned with genes, proteins, or epigenetics. To investigate the potential mechanism mentioned above, it needs comprehensively taking changes of physiology and biochemistry at all levels into account and managing them with the help of multi-disciplinary like TCM diagnosis science. TCM constitutions theory, imaging science, and so on are needed. Data harvested from TCM cohort consist of diverse data types, including information about pulse, tongue, face, smell, and inquiry, disease history, TCM constitutions, and many other types, whose analysis and reading require the assistance of machine learning. On the other hand, wearable equipments, such as effective phenotyping measurements, will be made to record the population’s health status and identify abnormal physiological markers, making the cohort survey convenient and precise. Various instruments can be used for TCM diagnosis, including the tongue diagnosis instrument (Li et al. 2021), pulse diagnosis instrument (Lan et al. 2020), and different kinds of TCM robots (Song et al. 2021). All these instruments will provide powerful hardware supports for the studies of Chinmedphenomics.

## Conclusion

In conclusion, Chinmedphenomics investigates potential TCM scientific mechanisms at different scales, which is personalized, precise and promising, providing novel insights into TCM studies in the future. Also, Chinmedphenomics may promote the phenomics progress, with the characteristics of Chinese wisdom and TCM theories.

## Data Availability

Data available on request from the authors.
